# Determination of Kennedy’s classification in panoramic X-rays by automated tooth labeling

**DOI:** 10.1007/s11548-025-03469-z

**Published:** 2025-06-24

**Authors:** Hans Meine, Marc Christian Metzger, Patrick Weingart, Jonas Wüster, Rainer Schmelzeisen, Anna Rörich, Joachim Georgii, Leonard Simon Brandenburg

**Affiliations:** 1 Fraunhofer Institute for Digital Medicine MEVIS, Max-von-Laue-Str. 2, 28359 Bremen, Germany; 2 Department of Oral and Maxillofacial Surgery, Medical Center – University of Freiburg, Hugstetterstr. 55, 79106 Freiburg, Germany

**Keywords:** Mask R-CNN, Tooth detection, Panoramic X-ray, Kennedy’s classification

## Abstract

**Purpose:**

Panoramic X-rays (PX) are extensively utilized in dental and maxillofacial diagnostics, offering comprehensive imaging of teeth and surrounding structures. This study investigates the automatic determination of Kennedy’s classification in partially edentulous jaws.

**Methods:**

A retrospective study involving 209 PX images from 206 patients was conducted. The established Mask R-CNN, a deep learning-based instance segmentation model, was trained for the automatic detection, position labeling (according to the international dental federation’s scheme), and segmentation of teeth in PX. Subsequent post-processing steps filter duplicate outputs by position label and by geometric overlap. Finally, a rule-based determination of Kennedy’s class of partially edentulous jaws was performed.

**Results:**

In a fivefold cross-validation, Kennedy’s classification was correctly determined in 83.0% of cases, with the most common errors arising from the mislabeling of morphologically similar teeth. The underlying algorithm demonstrated high sensitivity (97.1%) and precision (98.1%) in tooth detection, with an F1 score of 97.6%. FDI position label accuracy was 94.7%. Ablation studies indicated that post-processing steps, such as duplicate filtering, significantly improved algorithm performance.

**Conclusion:**

Our findings show that automatic dentition analysis in PX images can be extended to include clinically relevant jaw classification, reducing the workload associated with manual labeling and classification.

## Introduction

Panoramic X-rays (PX) are commonly used for routine diagnostics in dentistry and maxillofacial surgery to depict the patient’s teeth and surrounding structures in one 2D image [1]. The devices used to perform PXs are affordable, easy to handle and thus widely accepted in both the clinical environment and in dental practices where they provide the examiner with a concise overview of the patient’s dental status [2].

When planning prosthodontic restoration of partially edentulous patients, the existing dentition should be registered and categorized. The Fedération Dentaire International (FDI) proposed one of the most common tooth numbering systems, which is widely used to express the type and location of existing teeth using two-digit numbers. Unambiguous labeling of each tooth builds the basis for a straightforward communication with other dentists and dental technicians during the therapeutic process. Given individual tooth statuses described in such a scheme, Kennedy’s classification categorizes the topographical distribution of abutment teeth in partially edentulous jaws. This helps to estimate the complexity of the required prosthetic restoration and should therefore be determined in advance of any prosthodontic treatment [3].

The numbering of teeth and the determination of Kennedy’s classification in partially edentulous jaws are prone to error [4]. Especially when facing heavy workloads, artificial intelligence (AI) has the potential to support the dental practitioner [5].

Convolutional neural networks (CNN) have redefined the state of the art for many image analysis tasks such as instance segmentation, which consists of detecting objects (like teeth) in an image, classifying each of them into one of multiple relevant types, and segmenting each individual instance. Mask R-CNN is a prominent region-based CNN method for detecting and classifying objects at varying sizes with an integrated segmentation of each detected object instance [6].

Various publications investigate the application of AI in oral and maxillofacial radiology, albeit on different datasets. A multitude of different CNNs were used not only to detect teeth, but also to find pathologies on PX. Labeling and segmentation of teeth builds the basis for advanced diagnostics and was also addressed before [7–13].

Very recently, Khurshid et al. [14] published the first work toward automating Kennedy’s classification. Unfortunately, their interpretation of Kennedy et al. [15] differs from ours, so that edentulous areas with pontics or implants are disregarded. Furthermore, no unique classification of jaws is performed or evaluated, the study rather investigates gap detection.

This retrospective study aims to investigate automated dentition analysis on PX of partially edentulous patients based on tooth labeling using a Mask R-CNN with appropriate post-processing. This architecture was chosen because it is one of the few that can segment individual teeth even if they overlap in PX and because it performs detection, numbering, and segmentation of teeth in one model. Based on this, we automatically determine the jaws’ classification according to Kennedy et al. [15].

## Methods

A retrospective study was conducted to evaluate the automatic determination of Kennedy’s classification of the upper and lower jaws based on detection, labeling and segmentation of teeth pictured in PXs using a Mask R-CNN, a subsequent post-processing algorithm, and a rule-based jaw classification.

### Patient selection and imaging

Patients who received a PX during routine diagnostics in the Department of Oral and Maxillofacial Surgery of the Medical Center of the University of Freiburg, Germany between 07/2011 and 11/2020 were reviewed. Approval for this retrospective study was obtained from the Ethics Committee of the University of Freiburg. All included patients were aged between 18 and 65 and gave informed consent for scientific use of their radiographic data. Patients with orthodontic appliances, fractures, osteosynthesis plates, piercings, or fully edentulous jaws were excluded. Patients with implant-supported prostheses or fully dentate jaws were included.

All PXs were acquired using the Vista Pano S dental imaging unit (Duerr Dental, Bietigheim-Bissingen, Germany) with the following parameters: 73 kVp, 15 mA, 9204 msec. PXs with severe motion artifacts were excluded from further evaluation.

### Data annotation and segmentation

As a prerequisite for model development and evaluation, 209 PX were carefully selected, diagnosed and annotated by a dentist with 4 years of experience (PW). The selected PX images were imported into the data curation platform CuraMate (formerly SATORI) developed by Fraunhofer MEVIS [16]. This software was customized to support an efficient workflow for registering all existing teeth in a preformed FDI grid and for segmenting each tooth or implant interactively. A spline-based tool was preferred for the initial contour, and a brush tool was used subsequently for efficient local corrections (“pushing” the contour in the desired direction). Each FDI position was labeled according to its status (“missing”, “tooth”, “implant”) with sub-categories describing the presence of supraconstructions, removable or fixed prostheses etc. Finally, the upper and lower jaws were classified according to Kennedy [15]. CuraMate supports workflow states, which were used to implement a peer review of each annotation by another clinician with 5 years of experience (LSB).

**Fig. 1:**
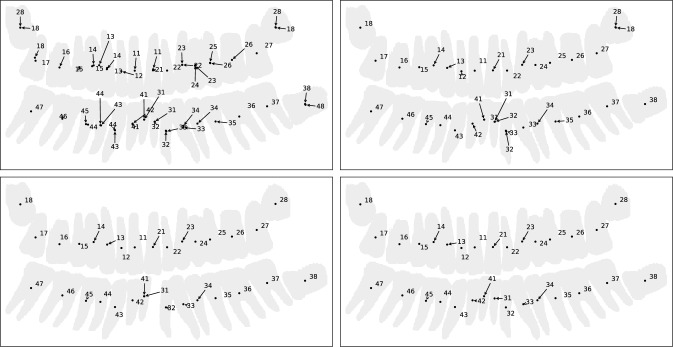
Post-processing of raw R-CNN output (top left) by filtering based on prediction score (top right), duplicate FDI position filtering (bottom left), and geometrical overlap checks (bottom right)

### Mask R-CNN-based instance segmentation

To be able to classify partially edentulous jaws, the dental status is first assessed using a Mask R-CNN [6], which is trained to perform instance segmentation and outputs a list of detected instances, each described with a bounding box, a classification label, a confidence score in the range 0...1, and a binary segmentation mask. As for Kennedy’s classification, implants, pontics and root residues should be disregarded, we used only annotations of potential abutment teeth to train the Mask R-CNN and used their FDI positions as corresponding classification labels. For training and evaluation, the data was split into five folds (on patient level, preventing biased evaluation), training five models each on three of the five folds (on average, covering about 125 cases) with a fourth fold used as validation data. Parameter decisions were made on the validation data, reserving the respective test data (fifth fold) for final evaluation.

The Mask R-CNN implementation was based on torchvision 0.12.0. Training was performed on NVIDIA GTX 1080 / 2080 Ti cards, 4 PX per minibatch, SGD with a momentum of 0.9 and a base learning rate of 0.001, performing validation every 80 iterations. Horizontally flipped images with appropriately adapted FDI position labels were used for data augmentation. The learning rate was halved when 10 validation rounds did not show any improvement of the $\text {AP}_{50}$ score anymore (average precision, measured at an overlap of 50% IoU). After five such decay steps, the training was stopped.

### Post-processing

The raw prediction of the Mask R-CNN still contains a number of duplicate instance, either from the same region proposal (with identical segmentation masks, but different classes), or from different region proposals that eventually resulted in strongly overlapping masks. In order to produce a final, cleaned result, we perform an elaborate post-processing that combines non-maximum suppression, a geometric overlap check, and also makes use of the fact that in our application, each FDI position can only exist once. Figure 1 illustrates each step that is described in the following.

***Filtering of instances with a prediction score*** $<0.5$

All instances are considered in order of decreasing prediction scores to check whether they should be discarded or selected for the final result. If the score is below a threshold, the predicted tooth instance is discarded. This threshold (which balances between sensitivity and specificity) is set to 0.5, meaning that the score for this FDI position must be larger than that of all other positions taken together. (An analysis on the validation set revealed very flat performance measure curves around this value.)

**Fig. 2:**
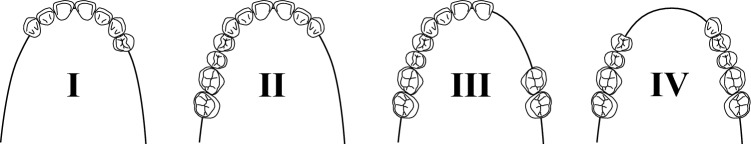
Kennedy’s classification of partially edentulous jaws [15]: I = bilateral shortened toothrow; II = unilateral shortened tooth row; III = no shortening of tooth row but gap; IV (special case of class III) = no shortening but gap across midline (multiple missing front teeth). Figure based on work by Kaligula, CC BY-SA 3.0

***Filtering of duplicate FDI positions***

Instances above this threshold are next checked for their predicted FDI position. If an FDI position is already occupied by a previous instance (with a higher score), later ones will be discarded. This “duplicate label filtering” is uniquely motivated by the tooth detection task. Note that this step relies on the predicted labels, so misclassification errors may result in erroneous filtering, as in the example illustrated in Fig. 1.

***Geometrical duplicate filtering based on segmentation***

Finally, we make use of the model’s segmentation output: When considering a new tooth candidate, we compute the fraction of tooth pixels in its segmentation mask that are “new” in the sense that they are not occupied by any previously selected instance. Since (contrary to popular street scene applications of the Mask R-CNN) the desired tooth segmentation masks often *do* overlap with neighboring teeth, the corresponding decision threshold is set to 50%. Again, the exact value of this parameter is not critical, since it only discriminates between very small overlaps between neighboring teeth and masks that cover the same teeth.

### Determining Kennedy’s classification

Our algorithm for the classification of jaws according to Kennedy et al. [15] (Fig. 2) first checks for the presence of the second molars (17 and 27 or 37 and 47): if both are missing, the class is initialized with I. If only one is missing, the class becomes II. It is set to IV if at least both front teeth (11 and 21 or 31 and 41) are missing while both second molars are present and the remaining rows of teeth are closed. If none of these conditions hold, but any other position (except wisdom teeth) is missing, the classification is set to III. Since not every jaw in our dataset is partially edentulous, sometimes none of Kennedy’s classes are applicable, and we assign a class “full” (17...27 or 37...47 all present, cf. Fig. 6a).

### Performance metrics

Our goal is to automatically determine Kennedy’s classification of partially edentulous jaws. For evaluating such classification problems, the confusion matrix provides a concise summary of all correct predictions and the different kinds of errors. Most other commonly used metrics can be derived from it.

Kennedy defines four types of *partially edentulous* jaws for prosthetic planning. Since we also want to handle fully dentate jaws, we computed 5x5 confusion matrices for the comparison of model based and expert assignment of Kennedy’s classification.

As Kennedy’s classification is based on the FDI labeling of detected teeth, confusion matrices of the underlying Mask R-CNN performing this task are provided as well. In addition to the 32 positions according to the FDI specification, it is possible that a tooth exists but is not detected (false negative, FN), or that a tooth is detected where it should not be (false positive, FP).

A prerequisite for this evaluation is to determine which reference tooth a given prediction corresponds to based on the geometric overlap. We consider a tooth detected if the Intersection-over-Union (IoU) between predicted and reference mask is at least 33% (corresponding to a Dice coefficient of 50%). Note that again, the exact value of this parameter is not critical, as 99.9% of all detections were found to have IoU values below 10% or above 60% on our validation data.

## Results

### Tooth labeling and segmentation

**Fig. 3:**
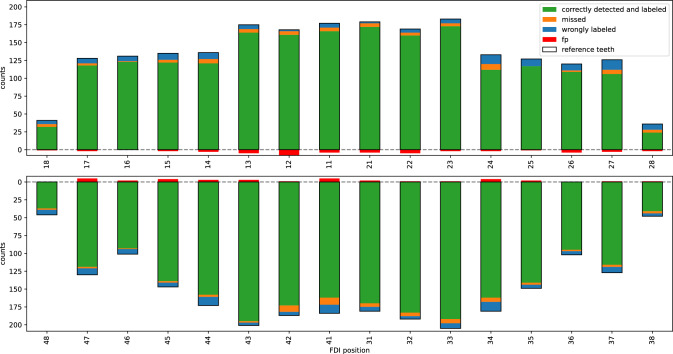
Counts of teeth by FDI position of the whole study group, as labeled by the examining dentist (PW). Colors indicate the fractions of the reference teeth that were correctly detected and the types of errors, respectively (see legend)

A total of 4,516 teeth in 209 PXs from 206 patients remained after labeling and peer review (see Fig. 3), which highlights the difficulty of our partially edentulous dataset (in total, 32.4% of all teeth missing). The canines were the most frequent type of tooth found in the presented study group (n=764; 91.4% prevalence), whereas wisdom teeth were missing in the majority of all subjects (n=171; prevalence 20.5%).

The overall training duration for the underlying Mask R-CNNs ranged 7000...7800 iterations in about four hours, corresponding to 215-245 epochs for each fold.

The resulting models detected 8,052 tooth instances, 5,172 of which had a prediction score above 0.5. The duplicate label filtering reduced the number of detected teeth to 4,646, and the full post-processing including the geometrical overlap check left 4,471 predictions. 4,156 of these were *correctly detected and correctly labeled* (93.0% of all predictions, or 92.0% of all reference teeth).

Of all types of teeth, upper incisors (n=659/693, 95.1%) and canines (n=724/764, 94.8%) were most often detected and labeled correctly. 83 teeth were *incorrectly detected* (FP) and did not overlap with the reference (with Dice $\ge 50\%$). Of all types of teeth, upper incisors (n=21/695 predictions; 3.0%) and molars (n=21/1124; 1.9%) were most likely to be FP. Out of the 4,516 annotated teeth, 130 (2.9%) were *not detected* (FN). Of all types of teeth, lower incisors (n=29/744; 3.9%) and molars (n=33/1136; 2.9%) were most often not detected.

**Fig. 4:**
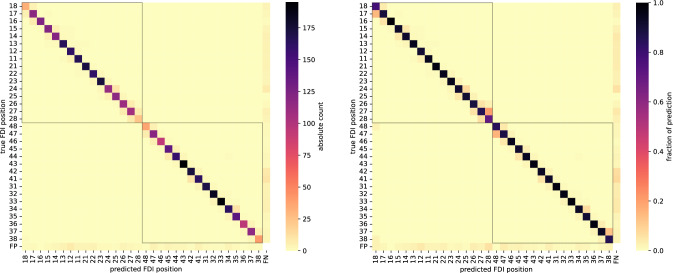
Evaluation of tooth labeling via confusion matrices (y-axes: reference labels by examining dentist; x-axes: model prediction). Off-diagonal values represent mislabeled teeth, false detections (bottom row), or missed teeth (rightmost column). The right matrix is normalized to better reveal errors in less frequent positions (i.e., molars)

In total, 232 correctly detected teeth were incorrectly labeled. This kind of error was primarily caused by the confusion of first, second and third molars (n=90/1137 molars; 7.9% of the reference), see Figs. 3 and 4.

### Kennedy’s classification

A total of 418 jaws were evaluated for the assignment of a classification. The *reference* labels contributed to Kennedy’s classifications as follows: I = 111, II = 81, III = 124, IV = 2, “full” = 100 jaws. We have verified that the manually assigned classes match the results of applying the rule-based classification to the reference tooth annotations, ensuring consistency of the manual annotations and verifying the implemented rules against our interpretation of Kennedy et al. [15].

**Fig. 5:**
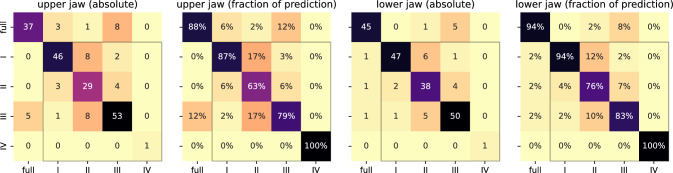
Evaluation of Kennedy’s classification (y-axes: true class (I-IV) or “full” for fully dentate jaws; x-axes: classification based on model prediction)

We applied the same classification rules to the post-processed predictions and assessed the correctness of the assigned classes against the above reference. In total, 347 (83.0%) jaws received the correct classification. The performance did not vary a lot between classes, but the results were slightly better for lower jaws (181/209 = 86.6%) than for upper jaws (166/209 = 79.4%), as illustrated by the confusion matrices in Fig. 5.

### Ablation studies on post-processing

As described above, our post-processing consists of three filtering steps—the score-based candidate filtering, a duplicate label suppression, and geometric overlap checks. In the following, we “ablate” (skip) these steps to measure their effect one-by-one. Note that these steps are not entirely independent, which could already be seen in Fig. 1: Filtering an instance whose pixel mask is already covered by previous detections may let another instance with the same label survive the duplicate label filtering.

***Ablation of geometric duplicate filtering***

Without the geometric overlap check based on tooth segmentations, the number of detected teeth that pass the filtering goes up from 4,471 to 4,646. As expected, sensitivity also rises, as the number of true positives (correctly detected, independent of label) rises from 4,388 to 4,562 at the expense of a single additional false positive (84 instead of 83). However, the labeling accuracy suffers (from 94.7% down to 92.4%), as the number of wrongly labeled teeth increases from 232 to 347 (meaning that 115 of the 175 additional tooth predictions are wrongly labeled).

On our test set, this results in many more misclassified partially edentulous jaws, with nine jaws less receiving their correct Kennedy class I, II, or III. On the other hand, eight more jaws are correctly classified as “full”.

***Ablation of duplicate FDI position label filtering***

Similarly, skipping the filtering of duplicate labels (predicted FDI positions) while keeping the geometric overlap check increases the number of predicted teeth from 4,471 to 4,568. True positives increase by 89 to a total of 4,477, and false positives increase from 83 to 91. Labeling accuracy drops to 92.8%, with 324 mislabeled teeth (+92).

The Kennedy classification results suffer most for “full” jaws (correct classifications decreasing from 82 to 73) and stay similar for partially edentulous jaws (+2, $+1$, $-1$, $\pm 0$ correct classifications for classes I-IV).

***Ablation of both geometric and duplicate position filtering***

With just the prediction score-based filtering left, the number of detected teeth rises to a total of 5,172, leading to a sensitivity of 99.4%. 5,059 of these even have good overlap with reference teeth, which shows that there are a lot of duplicate predictions, since the reference only contains 4,518 teeth. In total, 755 of these detections carry wrong or duplicate labels (16.7%). Additionally, there are now 113 false positives.

However, without the geometric duplicate filtering, the missing duplicate label filtering does not affect the Kennedy classification, since duplicate labels are just counted as “tooth present” and do not make a difference for the jaw classification. Hence, ablating both steps gives the same classification as just skipping the geometric filtering.

***Ablation of prediction score threshold***

It would be very uncommon not to filter out predictions with very low confidences. The total number of unfiltered predictions on our test data is 8,052, of which 2,880 instances have scores below our threshold of 0.5. It is interesting to note, however, how many of these instances are also removed by the two above filtering steps, even if no score-based filtering is applied: The two subsequent filtering steps are able to reduce the number of wrongly labeled teeth from 3,177 down to 249, and the number of false positives from 321 to 137. In fact, the overall accuracy of Kennedy’s classification is not affected (still 83%, although 3 upper jaws less are correctly classified as II, 3 more correctly receive class I).

## Discussion

**Fig. 6:**
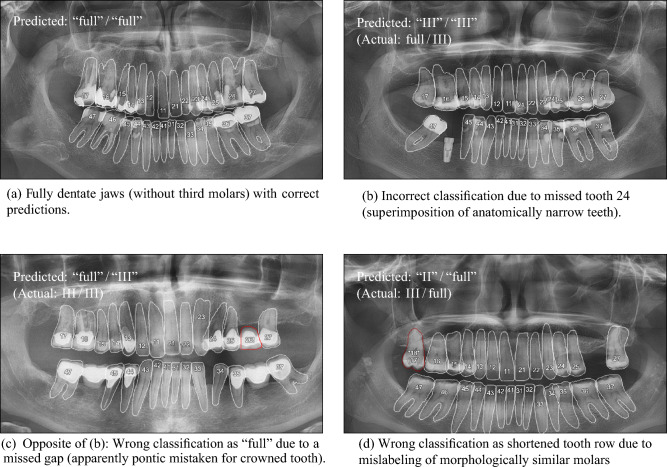
Typical error modes of the proposed method on our dataset

Kennedy’s classification was successfully automated with a simple rule-based system based on the detection and labeling of teeth in PX, for which the Mask R-CNN appears to be a viable tool, although it produces a considerable number of duplicate detections that motivated our post-processing. Kennedy’s classification was correctly predicted in the majority of cases (83%) and suffered most from mislabeled molars (Fig. 6d). The automatic determination of Kennedy’s classification was only partly addressed before [14], so there is no previously published accuracy to compare against.

Our underlying Mask R-CNN with post-processing has a good tooth detection performance (sensitivity 97.1%, precision 98.1%, specificity 96.2%) and assigns the correct FDI position to 94.7% of correctly detected teeth. Considering errors, our algorithm is more likely to miss existing teeth (2.9% of all reference teeth were FN) than to add false teeth (1.9% of all detected teeth were FP, e.g., due to artifacts, superimposition, or implant-supported pontics that should not be detected because they do not count as teeth for the purpose of Kennedy’s classification). However, the most common error was the confusion of (morphologically similar) teeth (5.2% of detections) or crowns and pontics (cf. Fig. 6).

Previous work had achieved high sensitivity (98%) and specificity (99%) for tooth numbering on specific datasets, e.g., by combining a Faster R-CNN for detection with a VGG-16 network for labeling [7]. Many other investigators also combine different networks to perform single tasks, requiring the subsequent application of multiple networks during inference. For instance, Mima et al. [8] combined a total of six Faster R-CNNs to label teeth in different subregions of PX.

To avoid confusion of morphologically similar teeth, an anchor-based object detection was proposed: Chung et al. [10] used Faster R-CNN and considered the anatomical order of teeth within the jaw (99% precision and 97% recall). However, in this work bounding boxes are created in all 32 FDI positions even with partially edentulous jaws, so that no statement about the topography of tooth loss is made.

According to a structured analysis [12], the commercially available AI tool “Apox” determines the presence of teeth and implants with a sensitivity of 95% and 84%, and a specificity of 90% and 99%, respectively. The results of our proposed workflow are comparable with the performance of this commercial software.

The comparison of existing works on tooth detection and labeling with our underlying algorithm is not only complicated by the fact that the exact tasks addressed by the authors differ, and that even similar tasks are evaluated with different metrics, but also that the datasets differ in size and in prevalence of teeth, pathologies, and implants. We can conclude, however, that in comparison, we provide an extensive evaluation on a relatively difficult dataset (on average, every third tooth missing) and used only a single model instead of multiple as in [7, 8, 11]. Also, our model outputs information on the presence of individual teeth (contrary to [10, 14]) and the segmentation output enables more future applications than just bounding boxes [7, 8, 10, 11] or image-level information [14].

### Limitations

The Mask R-CNN does not consider the absolute position of instances within the image. Instead, it primarily assigns FDI positions by appearance. Morphologically unique teeth (e.g., canines) were therefore less frequently mislabeled, and morphologically resembling teeth (first, second and third molars) were affected more.

Network architectures that take positions or even spatial relations into account may show better results in identifying teeth. One popular approach that could also deal with the many missing teeth in our dataset would be the Transformer. However, it requires a much larger amount of data and could not be trained on our dataset, with only about 125 images per training split to learn the geometrical relations from. Tooth migration could also lead to altered tooth positions within partially edentulous jaws.

The trajectory of the PX device that unfolds the dental arches into a two-dimensional image never exactly matches the anatomy. Blurry and indistinct depiction of teeth due to bad alignments may have contributed to the higher portion of FN for lower incisors and premolars.

The relatively low number of radiographs selected from the clinical database and annotated with segmentations may have introduced some unknown selection bias that we cannot quantify. Furthermore, the manual annotation process involved only two clinicians from the same group (annotator and reviewer), which might have introduced a bias, for instance when making the decision between root residue and (broken) tooth.

## Conclusion

For the first time, we successfully automated Kennedy’s classification of partially edentulous jaws (with 83% accuracy) based on automatic segmentation and labeling of teeth in panoramic X-rays. For the latter task, this study confirms Mask R-CNN to be effective even on our challenging dataset that includes many dental prostheses, implants, and missing teeth. We demonstrate that our proposed post-processing steps, including duplicate filtering based on FDI position and geometric overlap, further improved performance on the tooth level, without significant effect on Kennedy’s classification. The most common remaining errors involved the mislabeling of morphologically similar teeth, impacting Kennedy’s classification in certain cases.

In the future, the presented method could be extended to address additional tasks such as determining supporting areas according to Eichner by combining the information obtained from upper and lower jaws.
